# Biodiverse Management of Perennial Flower Margins in Farmland: Meandering Mowing by ‘Three-Strip Management’ to Boost Pollinators and Beneficial Insects

**DOI:** 10.3390/insects15120953

**Published:** 2024-11-30

**Authors:** Laurian Parmentier, Hannah Vanderstappen, Geert Haesaert

**Affiliations:** 1 Agrozoology Lab, Department of Plants and Crops, Faculty of Bioscience Engineering, Ghent University, Coupure Links 653, 9000 Ghent, Belgium; 2 Department Plants and Crops, Faculty of Bioscience Engineering, Ghent University, Valentin Vaerwyckweg 1, 9000 Gent, Belgium

**Keywords:** perennial flower margins, three-strip management, meandering mowing, spatio-temporal variation, Hill numbers, plant–pollinator networks, pollinators, beneficial insects

## Abstract

Agricultural intensification has led to significant declines in beneficial insect populations, such as pollinators and natural enemies, along with their ecosystem services. The installation of perennial flower margins in farmland is a popular agri-environmental scheme to mitigate these losses, promoting biodiversity, pollination, and pest control. However, outcomes can vary widely, and recent insights into flower margins in an agricultural context suggest that management could be an important contributor to this variation. This study evaluated two mowing management regimes: the new “three-strip management” method with uneven, curved mowing lines and regular phased mowing as a control method. During the third year of application, we evaluated the effects on the alpha diversity indices of pollinators and natural enemies, as well as plant–pollinator visitation networks. Curved three-strip management significantly increased the abundance of all pollinator groups (+44%) and natural enemies (+50%), and the taxonomic richness and diversity of pollinators, especially for rarer solitary bees. Floral diversity was also higher, with more unique plants blooming in early spring and late summer, generating more unique plant–pollinator interactions (+54%) and a positive impact on multiple network-level properties. Our findings provide new evidence that nature-based management methods can be a win–win solution, creating high-quality habitats that enhance the insect diversity of various groups, support associated ecosystem services, and help restore overall farmland biodiversity.

## Simple Summary

Intensive farming has caused a sharp decline in beneficial insects like pollinators (such as bees and butterflies) and natural predators of crop pests. One way to address this is by sowing flower margins on farmland, which can boost biodiversity and the numbers of beneficial insects for pollination and pest control. However, after installation, the success of these margins can vary based on how they are managed. In this study, we compared two mowing management methods: the new “three-strip management” method with variable, curved mowing lines and a traditional, regular phased mowing method using straight mowing lines. During the third year of application, we measured how each method affected insect diversity, floral diversity, and plant–pollinator interactions. Our results showed that three-strip management significantly increased the abundance and diversity of pollinators, especially bees and butterflies, and the abundance of natural enemies. Additionally, we found more plant species blooming at different periods of the year and a positive impact on multiple network properties of plant–pollinator visitations in the three-strip-managed margins. This study shows that new, nature-based management methods can enhance insect diversity, improve their beneficial function in the ecosystem, and contribute to overall farmland biodiversity.

## 1. Introduction

Insect populations are currently experiencing dramatic declines reported at global, regional, and local scales, notably in anthropogenic landscapes such as farmland. As a striking example, Hallmann et al. [1] reported a 75 percent decline over a 27-year period (between 1989 and 2016) in the total biomass of flying insects in Germany. Generally, the observed decline can be attributed to various environmental changes, including agricultural intensification, changes in landscape structure, and climate change [2,3,4], leading to uniform and flower-poor habitats. This has negative repercussions on insect populations, notably on beneficial insects such as pollinators and natural enemies. Because these insects provide important ecosystem services, including the pollination of wild plants and insect-pollinated crops and the predation of crop pests, their decline is deeply alarming due to the negative effects on such free services [5,6].

In farmland, the decline of flower-rich habitats is strongly linked to agricultural intensification [7,8]. Over the past half-century, it has been shown that habitat diversity and floral resources were reduced when farms became more specialized, leading to larger uniform fields instead of mixed farming on a smaller scale [9,10]. As an example, in the UK, there has been a 65% decline in the number of farms since 1945. In addition, the application of nitrogen-rich fertilizers has led to those plant species better adapted to nutrient-rich soils predominating, especially grasses, while the more characteristic and rarer dicotyledons have continued to decline [8,9,11,12]. As a consequence, in modern agricultural landscapes, semi-natural habitat elements like field margins have been severely reduced, although they provide essential resources to pollinators and many beneficial insect species [3,12,13,14,15,16]. Thus, the remaining semi-natural habitats suitable to supporting diverse insect populations have become increasingly scarce, are small and isolated, are often of poor quality, and are therefore predominantly utilized by generalist species. In relation to pollinators such as bees and butterflies, for example, studies have shown an overall decline in wild bee richness and abundance [17] and a lack of specialist butterflies on farmland [18].

To counteract this trend, flower margins are increasingly being installed as a popular agri-environmental measure (AEM), and are even being optimised in flower compositions [19,20], to foster diverse beneficial insect populations [13,21,22,23,24]. Such a wildflower habitat is aimed at providing pollen and nectar resources, alternative prey, and refuge from frequent disturbance, which are best obtained through the installation of perennial flower margins [25,26]. However, despite these efforts and the demonstrated benefits to many species, perennial margins often fail to meet their goals over time due to degradation in an agricultural setting. For example, Albrecht et al. [27] found that wild bee and hoverfly abundance sharply declined two years after the establishment of perennial margins in agricultural landscapes in Switzerland. Often, the generally nutrient-rich conditions of farmland soils have a negative effect on the growth of flowering dicotyledons over grasses [11,12,24]. In addition, inadequate management, such as the application of flail mowing with a conditioner (see, e.g., [28]), does not counteract this effect and rather worsens the margins’ status, making them less suitable habitats for pollinators and beneficial insects.

To address the mowing management of perennial flower margins, more effective schemes typically involve the mowing and removing of clippings to mitigate succession and prevent the margins from turning into uniform grass areas with few remaining flowering perennials [24]. However, in such schemes, it is often the case that the entire margin is mown at once, which can negatively affect beneficial insects that are sensitive to the destructive impact of mowing [29,30]. Some schemes divide the mowing zone spatially into two cycles, with the timing based on local guidelines or regulations (e.g., first cut in mid-June–early July and a second cut in mid-September–early October) [31]. Phased or rotational mowing, where rectangular strips are mown at different times, is preferable to mowing the entire margin simultaneously. Despite this, phased mowing schedules often overlap spatially, leading to the entire margin being mown over the course of a year. Hence, during one year’s seasonal cycle, few or no unmown zones remain in the margin. Unmown zones, which consist of taller plants and forbs, develop from senesced flowers and grass-like plants. These areas contribute to both biotic and abiotic variation in the margin, including microclimates, and provide essential habitat elements like shelter and nesting and mating places, such as for wild bees [32]. These overwintering structures are crucial in supporting a variety of insect groups, especially when combined with a variety of flowering dicotyledons. A study on meadow butterflies found that both nectar availability and the shelter provided by tall, overwintering vegetation were key factors in insect diversity along roadside grass margins [33]. Moreover, management can have an impact on plant–pollinator mutualisms [3], impacting network structure and stability, as has been reported for grassland habitats [34,35].

As studies report flower abundance and pollinator diversity declining after the establishment of perennial margins in intensive farming areas, and suggest regular reseeding as a solution [8,27], new management approaches may offer better alternatives. To improve habitat quality, especially in agricultural landscapes, a novel management technique for perennial margins has recently been introduced by Parmentier, called “three-strip management” [36]. This method uses curved, uneven mowing lines (instead of straight ones) to create diverse mown and unmown areas after each mowing cycle that serve as shelter for fauna [37]. With each subsequent cycle, a new curved line is used that intersects with the previous one, maximizing irregularity in the mowing pattern. Because the mowing lines differ with each mowing cycle, some zones remain unmown while others are mown once or twice throughout the season. This results in enhanced habitat complexity and promotes prolonged flowering and varied conditions for insects during the season and over years. When tested in a two-year pilot study, the new mowing method showed positive effects on bee diversity by the second year [36].

In this study, we build on the 2023 study by Parmentier to further evaluate the effects of three-strip management after two years, i.e., during the third year of application on perennial flower margins, and compare it to regular phased mowing. This study aims to assess the impact of management on the alpha diversity metrics (abundance, Hill numbers) of different insect guilds, focusing on pollinators (bees, syrphids, butterflies) and the natural enemies of crop pests. Additionally, the impact of management on plant–pollinator visitation networks is assessed. We expect that the initial positive effects of three-strip management reported on pollinating bees will be maintained or even enlarged during the third year of application and that this novel management will have a positive impact on the average value of bipartite properties of plant visitation networks. Secondly, we expect that the effect can be broadened to other insect groups, particularly to natural enemies, benefiting them more compared to regular phased mowing management.

## 2. Materials and Methods

### 2.1. Study Sites and Perennial Margins

**Figure 1:**
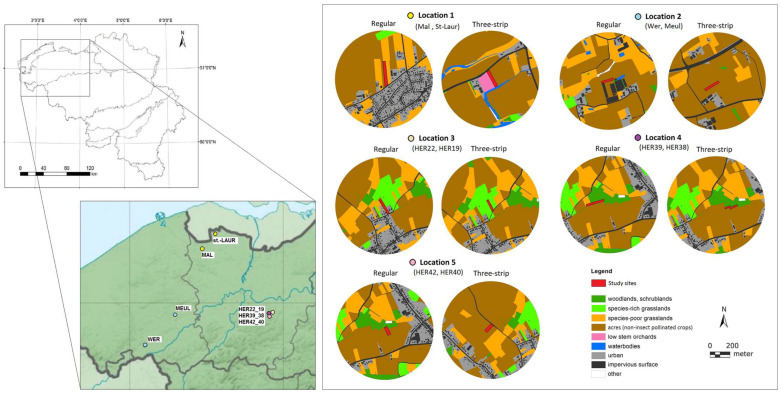
Selected locations in Flanders (Belgium) and paired sites (indicated with a unique color) of perennial flower margins (in the center, indicated in red) with either three-strip or regular management, and surrounding land cover (within a radius of 500 m) mapped for each study site.

Five study locations were selected in the provinces of East and West Flanders (Flanders, Belgium), and in each location, two paired study sites (in total, ten sites) were established, as shown in Figure 1. Study sites selected within one location were of equal size and consisted of two perennial flower margins (width of 10 ± 5 m and length of 100 ± 40 m). Flower margins were primarily situated in an agricultural environment, surrounded by a landscape matrix of small landscape elements (SLEs). These SLEs included hedgerows, solitary trees or tree rows, and gardens, as well as paved elements such as streets and buildings.

Prior to the start of the experiment, we performed a landscape metric analysis to ensure that the surrounding landscapes of paired study sites within a location were similar. Land cover data were retrieved from the Biological Valuation Map (BVM) of Flanders, Belgium [38], and analyzed in QGIS [39]. BVM categories were grouped into seven land cover categories based on the resources they provide for pollinators and beneficial insects. The following land cover categories were identified: (1) “Semi-natural positive”, including all semi-natural habitats (e.g., permanent grasslands, woodlands, positive linear elements and small landscape elements (SLEs), permanent well-managed grassland strips flanking road verges and riversides) that can provide food or nesting opportunities; (2) “Species-poor grasslands”, including all grasslands that do not provide food or nesting (e.g., species-poor temporary grasslands, species-poor permanent grasslands, linear poor elements such as frequently mown lawns); (3) “Urban”, defined as the percentage of built-up areas, industrial areas, and roads based on the Large-scale Reference Database of Flanders; (4) “Acres (non-insect-pollinated crops)”, including all agricultural fields that produce crops that do not rely on pollinators (e.g., grain, potatoes, maize, beets); (5) “Low-stem orchards”, describing all plantations of low-stemmed fruit trees (e.g., apples, pears, sweet cherries), characterized by intensive management; (6) “Waterbodies” (e.g., ponds, brooks, rivers); and (7) others, encompassing undefined elements in the land cover maps (a minority portion of the full land cover). We ensured that the surrounding landscapes of paired study sites within a location were similar. An assessment of landscape variation in the study sites relative to the management factor is given in Supplementary File S1.

In the winter of 2019–2020, margins were sown with perennial flower mixtures from the BEESPOKE project [40] (Supplementary Table S1). By the third year, the margins primarily contained flowers with a small amount of naturally occurring grasses (Poaceae) that were not part of the seed mix. Initially, in 2020, all margins were managed with regular phased management and regarded as fully developed before they were divided into one of the two management groups. This phased mowing involved cutting half the margin in spring (late June to early July) and fully mowing at the end of summer (mid- to late September). Starting in 2021, paired margins were managed according to their designated management method.

### 2.2. Mowing Management Methods

**Figure 2:**
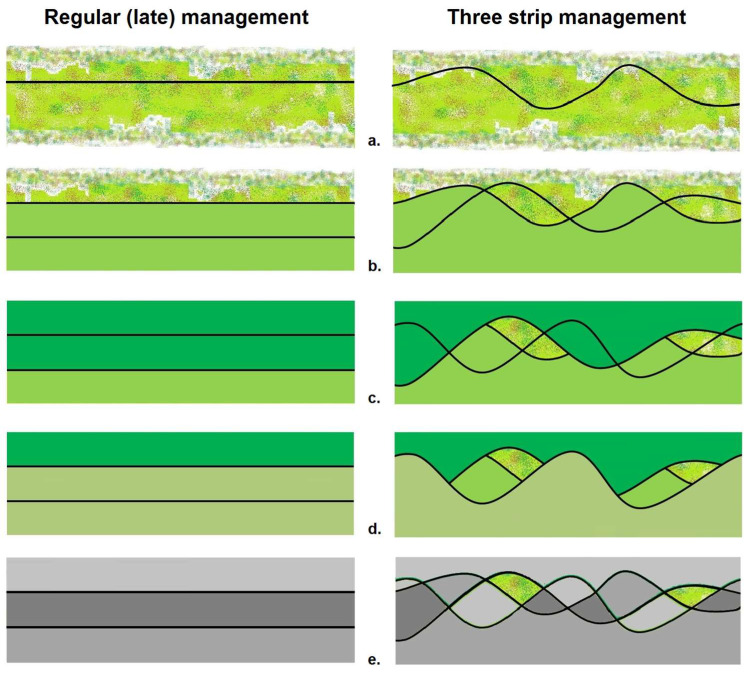
Illustrating the combined effect of three successive mowing cycles on a margin when regular versus three-strip management is applied, according to Parmentier (2023) [36]. (**a**). The first mowing line, starting from an unmown margin. The margin is longitudinally divided into three equal strips, of which one-third will be kept unmown (ratio of mown/unmown is set at 2:1). When applying three-strip management, a curved instead of a straight mowing line is used. (**b**). The result after the first mowing cycle in the first season (uniform green zones are mown), normally applied at the end of June, and an illustration of the second mowing line. (**c**). The result after the second mowing cycle, normally applied at the end of September in the same season, and an illustration of the third mowing line. (**d**). Result after the third mowing cycle, the first of the second season. (**e**). Overlay illustrating the effect after three mowing cycles when either regular or three-strip management is applied, respectively (unequally mown zones in different grey tones; unmown zones in green).

Applying a paired study design, two perennial flower margins at each location were managed using either three-strip management or regular management. Briefly, the novel three-strip management method, following Parmentier (2023) [36], is applied in different steps, as shown in Figure 2, for three successive mowing cycles (i.e., for example, two cuts in the first year and one in the year after). To implement the new three-strip management method in a season with two cuts (one cut in mid-June–early July and a second cut in mid-September–early October), the margin is first divided into three longitudinal strips, hence the name. Then, two variable curved cutting lines are applied between the inner borders of the imaginary strips, meandering around the imaginary (dashed) lines and ensuring a consistent 2:1 ratio of mown to unmown areas. In the field, these curved mowing lines represent the two mowing cycles in the season. Here, a key element is the mowing lines crossing each other at some points, to generate spatial variation in (un)mown zones. It is also important to mention that the cut and uncut parts defined by the curved mowing line should be alternated with each mowing cycle, with the process repeated each year [36].

Regular late mowing (or phased) management involved cutting two-thirds of the margin in rectangular zones (longitudinal direction), leaving one-third unmown. The mown and unmown zones were alternated with each cycle, also maintaining a consistent 2:1 ratio, and the process was repeated annually.

Across all sites, the same mown-to-unmown ratio, a cutting height of approximately 10 cm (4 inches), and the use of rotary mowers (1.5–2.5 m width) were applied, with clippings being removed after each mowing cycle. While the mown and unmown zones within each study site were alternated with each mowing cycle, over the course of one season under regular management, the entire margin was mown, while three-strip management typically retained variable unmown zones. Thus, basically, the effect of uneven curved versus straight mowing lines was tested, with all other parameters held constant.

### 2.3. Landscape Analysis

To assess the impact of landscape on the study design before starting the experiment, a PERMANOVA analysis was conducted using detailed land cover data within a 500 m radius of each study site, with landscape metrics categorized by their attractiveness to pollinators (Supplementary File S1). The explanatory variables in the PERMANOVA were location (five levels) and period, with management type (two levels: three-strip and regular management) as the grouping (or strata) factor. Categorical data were first transformed with a centered log-transformation using the decostand function to obtain a similarity matrix based upon Euclidean distance, and a permutational multivariate analysis of variance (PERMANOVA) was performed with the ‘adonis’ function in the R package “Vegan” [41]. Data analysis and all further analysis were performed in R vs. 4.1.3 [42].

### 2.4. Pollinator and Floristic Surveys

The monitoring of beneficial insects in this study focused on pollinators, including honeybees, bumblebees, solitary bees, all day-flying butterflies (Papilionoidea), and natural enemies selected for their beneficial predation effect on crop pests.

Insects were monitored using area–time counts over the entire margin with a fixed duration, and each survey consisted of 2 rounds of 30 ± 5 min effective monitoring conducted on the same day, one before and one after 1:30 p.m. All monitoring was conducted between 10:00 a.m. and 6:00 p.m. [43,44] during appropriate weather conditions (low wind speed, temperature above 18 degrees, and not on cloudy days). During the third year of the mowing management regime in 2023, four to five survey rounds were conducted in each location. Monitoring rounds were spread between mid-April and mid-September, and were always executed in units of paired study sites. Rounds were chosen randomly over the full margin and within each location.

For each individual pollinator (bees, butterflies, syrphids) observation, the flowering plant was also recorded. The species of pollinators and natural enemies were determined in the field if possible, or specimens were put in coded tubes for identification at the species level in the lab. Cryptic bee species, such as those under the *Bombus terrestris* complex, were aggregated and included *B. terrestris*, *Bombus lucorum*, and *Bombus cryptarum*. During each monitoring round, the abundance of flowering plants was scored at the margin level using the Tansley scale estimation method [45].

### 2.5. Plant–Pollinator Visitation Networks and Analysis

To assess the effect of mowing management on plant–pollinator network properties, individual pollinators visiting flowering plants were recorded and plant–pollinator visitation networks were constructed. For this analysis, we only included a subset of pollinator species that were obligate flower visitors [46]. Flower visits were pooled by pollinator group (bees, syrphids, butterflies), monitoring period, and location according to management type. Plant–pollinator visitation networks per pollinator group and management type were calculated using the ‘bipartite’ package in R. For each management type, network-level properties per location and monitoring period (n = 4) were calculated separately, to avoid bias or the creation of forbidden links [47]. Network properties were averaged values across periods and locations (total n = 16). Based on insights reported in the recent literature [46,47,48], the following network-level parameters were calculated [49]:

- N interactions: number of plant–pollinator interactions;
- Fisher’s α: measure of network interaction diversity;
- Links per species: mean number of links per species in the network;
- Weighted connectance: realized proportion of possible links, weighted by network size;
- H_2_’: network generalization;
- Togetherness (plant level): mean number of co-occupancies across all species combinations;
- Extinction slope (pollinator level): simulated secondary loss of pollinators with extinctions of species at the plant level;
- Robustness (pollinator and plant level): “fragility” of a level to losses at the other level;
- Functional complementarity (pollinator and plant level): extent of sharing of interactions between plants and pollinators, respectively.

### 2.6. Data Analysis and Statistics

The effect of management type on assemblages of pollinators and natural enemies was investigated, with a focus on their abundance (counts, N) and diversity indices at the species level, i.e., species richness (Hill number N0, ^0^D), exponential Shannon diversity (Hill number N1, ^1^D), and inverse Simpson diversity (Hill number N2, ^2^D). Here, index ^1^D (Hill number N1) captures the contribution of rare species to overall diversity, while index ^2^D (Hill number N2) accounts more for common species in a community [50,51]. All diversity indices based on Hill numbers were calculated using the package ‘HillR’ in R [51,52]. Network-level properties were calculated using the ‘bipartite’ package in R [53]. We applied (generalized) linear mixed models (GLMs, GLMMs) to analyze the effects of management type on the total abundance (N) and alpha diversity indices (species richness, S (^0^D), exponential Shannon diversity e^H’^ (^1^D), and inverse Simpson diversity D_2_ (^2^D)) of pollinator groups (bee, butterfly, syrphid, combined) and natural enemies (combined), and the effects of the different plant–visitor network-level indices for pollinators (bees, syrphids, and butterflies separately). The diversity indices e^H’^ and D_2_ and the network-level properties were modeled with the assumption of a Gaussian distribution. For the other response variables, we tested several statistical distributions that are suitable for discrete, positive data (Gaussian, Poisson, negative binomial, and quasi-Poisson). Model fits were confirmed by checking residual plots and—in case of the distributions for discrete data—checking for overdispersion using the dispersion test in the AER package [54]. In cases where overdispersion was detected, we corrected the standard errors using quasi-Poisson GLMMs [55]. We used the Akaike Information Criterion (AIC) to select the best model [56]. Transects nested at locations were included as random intercepts. We tested the effect of management type, survey period, and their interaction as explanatory variables. Final best models were also checked against zero-inflation, autocorrelation, overdispersion, and collinearity effects using the “performance” package. All GLMM analyses were carried out using the package “lme4” ver. 1.1–21 for Poisson GLMMs [57] and “glmmTMB” ver. 0.2.3 for quasi-GLMMs, with “Nbimon1” or “Nbimon1” family for quasi-Poisson GLMMs [58]. Post-hoc Tukey tests were performed on appropriate models which had a significant result. All figures were created with the ggplot2 package [59].

## 3. Results

### 3.1. Landscape Analysis

The landscape analysis (Supplementary File S1) shows that the study sites (management factor) within the selected locations do not significantly differ (F_5,10_ = 0.963, *p* = 0.489), and locations are situated in landscapes with equal metrics (F_4,5_ = 0.981, *p* = 0.565). Thus, within locations, the selected study sites show no differences with respect to the landscape context, justifying our paired study site design and confirming that in the experimental setup, surrounding landscapes have a similar impact on insect groups monitored in perennial margins to study sites.

### 3.2. Species-Level Effect of Management on Pollinators and Natural Enemies

Over a full monitoring season (between April and the end of September) a total of 2933 bees, 1681 hoverflies, 753 butterflies, and 1131 natural enemies were observed across the flower strips. Monitoring results are listed in Supplementary Table S2.

#### 3.2.1. Pollinators

In the three-strip-managed flower margins, 58% of all observed *visits* of bees, 60% of all observed visits of butterflies, and 60% of all observed visits of hoverflies were recorded, an increase of 38%, 44%, and 50%, respectively, compared to regular management. A total of 45 bee species were identified, with 39 species at the three-strip-managed sites and 25 at the regular-managed sites. Several species on the Belgian Red List of Bees were observed, mostly in the three-strip-managed sites, including *Sphecodes spinulosus* (three-strip), listed as critically endangered; *Lasioglossum brevicorne* (three-strip), listed as endangered; *Bombus jonellus* (vulnerable, three-strip); and *Bombus campestris* (vulnerable, regular). *Bombus pascuorum* was most frequently observed at all sites, representing 41% of bee visits, followed by *Bombus lapidarius* (19%) and *Apis mellifera* (18%). Solitary bee species accounted for 55% of observed bees in the three-strip margins and 51% in the regular ones. The families Halictidae, Colletidae, and Megachilidae were most common across both management types.

Analyses (GLMMs) of alpha diversity indices (Table 1) showed a significant positive effect on the number of bees (N) for margins with three-strip management compared to regular management (SE = 0.129, Z = −2.481, *p* = 0.013). We also found a significant positive effect on species richness (S: SE = 0.113, Z = −3.182, *p* = 0.002) and exponential Shannon diversity (e^H’^: SE = 0.319, Z = −2.843, *p* = 0.004), while the effect on inverse Simpson diversity was significant but less pronounced (D_2_: SE = 0.242, Z = −2.210, *p* = 0.027). Interestingly, when focusing on solitary bees analyzed as a subcategory, significance levels are more pronounced for the three-strip-managed sites (*p* = 0.001), while only a borderline significant effect was observed for the more common social bee species (*Bombus* spp. and *A. mellifera*) (*p* = 0.048), an observation that is in agreement with the differences noticed in effect levels between the indices e^H’^ and D_2_, representing greater effects on rarer bees compared to more common bee species, respectively.

For butterflies, 19 species were identified, with 18 species at three-strip-managed sites and 15 at regular sites. Pieris rapae (32%) and Maniola jurtina (22%) were the most abundant species, particularly in the three-strip-managed sites. Total butterfly abundance was significantly higher in margins with three-strip management (SE = 0.289, Z = −3.028, *p* = 0.002), where higher effects on the different diversity indices were also observed (S, e^H’^, D_2_: all *p*-values < 0.003).

A total of 30 syrphid species were observed, with 23 species in the three-strip-managed margins and 22 in the regular-managed flower margins, resulting in a 50% increase in syrphid abundance with three-strip management. Sphaerophoria scripta (39%) and Eristalis tenax (24%) were the most common species across all sites, regardless of management type. The analysis showed significantly higher syrphid abundance (SE = 0.16, Z = −2.021, *p* = 0.043), but no significant effects were found on diversity indices (Table 1).

Overall, combining all pollinator observations, the three-strip-managed margins showed a significant increase in total abundance (SE = 0.112, Z = −2.92, *p* = 0.003), species richness (S: SE = 0.573, Z = −4.749, *p* < 0.001), and exponential Shannon diversity (e^H’^: SE = 0.436, Z = −3.007, *p* = 0.001), while there was a less pronounced significant effect on inverse Simpson diversity (D_2_: SE = 0.334, Z = −2.351, *p* = 0.019).

#### 3.2.2. Natural Enemies

Of the total number of natural enemies observed, 62% were found in the three-strip-managed margins. The family Syrphidae formed the largest subgroup, accounting for 77% of the total number of natural enemies (867 observations), followed by the Coccinellidae family (87 observations, of which 48 were found at the three-strip-managed sites). *Coccinella septempunctata* was observed most frequently, with 61 observations. Also, Ichneumonidae spp. were encountered frequently, with 49 individuals in total (Supplementary Table S2). Statistics on these observations showed that the number (N) of beneficial insects was significantly higher at the three-strip-managed sites (SE = 0.129, Z = −3.266, *p* = 0.002). In addition, no significant differences were observed in terms of species richness (S) and diversity indices (e^H^ and D_2_), while for e^H’^ (Hill number N1), which represents the rarer species in the community, a borderline significant effect was missed at alpha = 0.1 (SE = 3.365, Z = 1.438, *p* = 0.105) (Table 1).

In addition, we performed an analysis of all beneficial insects observed in this study (pollinators and natural enemies pooled together) per management type. In this analysis, all indices, i.e., numbers (N), species richness (S), and diversity indices (e^H^ and D_2_), were significantly better in the three-strip-managed margins, with strong significance levels (all *p*-values ≤ 0.005) (Table 1).

### 3.3. Effect of Three-Strip Management on Floral Composition

**Figure 3:**
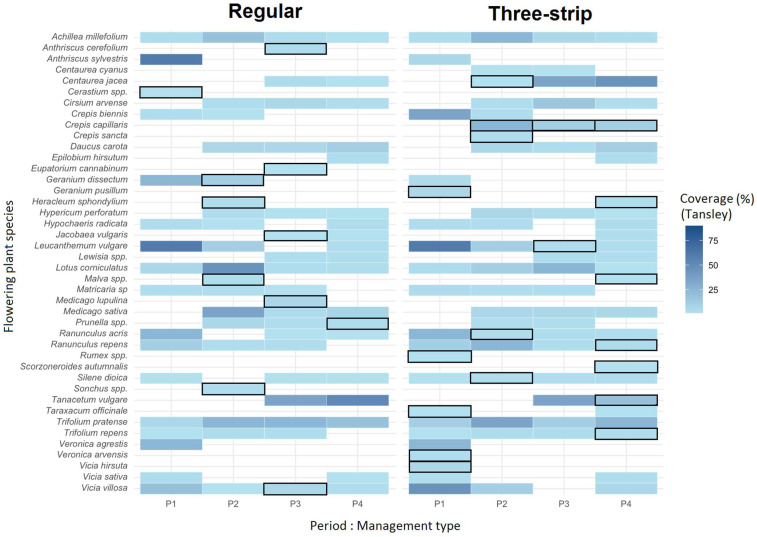
Plant species coverage for regular versus three-strip management. Blue elements represent individual plant species coverage (%) based on full study site estimations (Tansley scale), averaged per period and management method. Blue color intensity represents the coverage percentage. Black framed elements highlight unique blooming plant species observed per management type during each period (P1–P4).

The abundance plot in Figure 3 visualizes the average abundance of observed flowering plants, per management type and period, over four periods, covering the entire foraging season (period 1: April–May; period 2: June; period 3: July–August; period 3: September). In total, 37 flowering plant species were observed across all periods in margins with three-strip management, while only 33 species were found in margins with regular management. In the latter, fewer species were present, and some were dominant or abundant, such as *Anthriscus sylvestris* and *Achillea millefolium*. In contrast, in the three-strip-managed margins, no dominant species were observed, and only a few abundant species were observed, including *A. millefolium*. Interestingly, some plant species that were very attractive to solitary bees, e.g., *Crepis capillaris*, *Crepis sancta*, and *C. jacea*, were found uniquely, or in higher abundance, in margins with three-strip management. When focusing on all unique flowering plant species (indicated with black frames in Figure 3), a comparison can be made for the four periods between the two management types. Here, when the spring (P1) and late summer (P4) periods are taken together, 12 flowering plants were found only in the three-strip-managed margins, outnumbering the regular-managed ones, where only two unique plant species were observed. In the late spring–summer period (P2–P3), with seven and nine unique observations in the three-strip-managed and regular-managed margins, respectively, the management effect is less pronounced.

### 3.4. Plant-Pollinator Network-Level Responses

#### 3.4.1. Plant–Bee Visitation Networks

**Figure 4:**
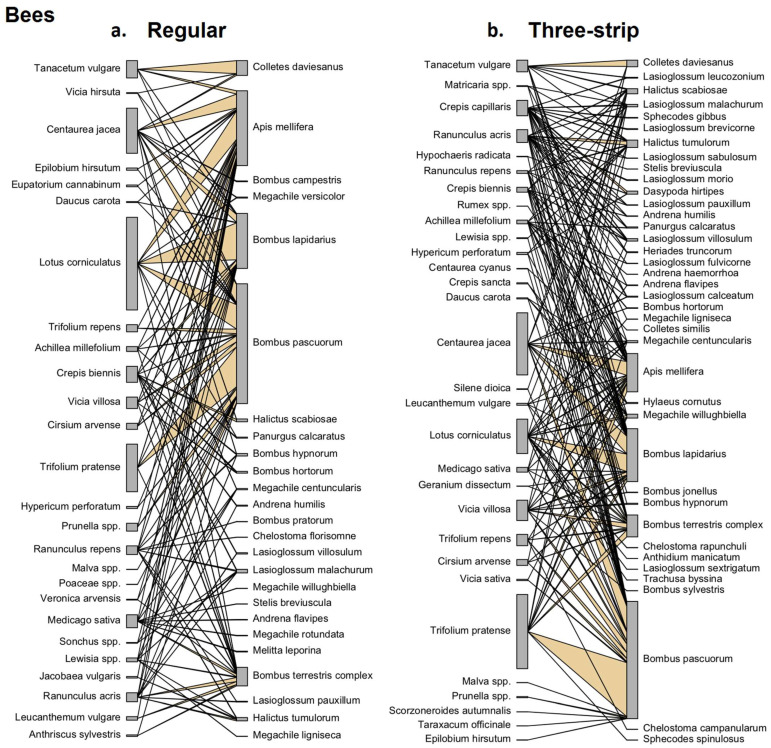
Plant–pollinator visitation networks. Visualization based on all bees observed on flowers during the third year of successive mowing, when applying regular rotational management (**a**). versus three-strip management (**b**). The most frequent flower–pollinator interactions and visits are highlighted in pale orange. The total number of plant–bee interactions was significantly higher within three-strip-managed sites; other networks and network properties are listed in Table 2.

Figure 4 illustrates the plant–pollinator visitation networks for all observed bees per mowing management method. It is clearly visible that the three-strip-managed sites have a higher total number of interactions, with more bee species also observed in the three-strip-managed versus regular-managed margins—25 species versus 40 species, respectively. A total of 112 unique interactions were recorded at regular-managed sites, compared to 173 at three-strip-managed sites. This difference was especially notable for solitary bees, with 35 interactions at regular-managed sites versus 93 at three-strip-managed sites. For solitary bees, most of the interactions observed were between *Colletes daviesanus* and *Tanacetum vulgare* in both management types, but other solitary bees were also commonly found on yellow composites (Asteraceae), such as *C. capillaris* and *C. sancta*, and on flowering plants from the Ranunculaceae family, such as *Ranunculus acris* and *Ranunculus repens*.

Significant differences in network properties between the management methods were also confirmed by bipartite network-level analysis (Table 2; N. interactions, GLMM, SE = 0.0378, Z = −9.00, *p* < 0.001), i.e., 301.50 ± 112.75 versus 423.25 ± 118.65 interactions (+41% in the three-strip-managed sites). Table 2 summarizes results for different network-level properties based on averaged values for plant–pollinator networks, with additional GLMM statistics comparing the network-level effects of the two management methods. For bees, the following network properties were found to be significantly different for the three-strip-managed margins: a higher Fisher’s alpha (SE = 0.0378, Z = −9.00, *p* < 0.001), more links per species (SE = 0.050, Z = −4.47, *p* < 0.001), lower weighted connectance (SE = 0.012, Z = 2.203, *p* = 0.027), lower network generalization, H_2_’ (SE = 0.092, Z = 2.884, *p* = 0.004), and higher robustness for pollinators (SE = 0.005, Z = −8.41, *p* < 0.001), as well as higher functional complementarity for plants (SE = 34.73, Z = −3.491, *p* < 0.001) and bees (SE = 40.37, Z = −3.575, *p* < 0.001). Also, for the parameter ‘pollinator extinction slope’, a measure of the simulated secondary loss of pollinators when plant species get lost in the network, a borderline significant effect was observed for three-strip-managed sites (*p* = 0.067).

#### 3.4.2. Plant–Butterfly Visitation Networks

A separate network analysis based on butterfly visitations per management type was performed (Supplementary Figure S1). For both management types, the most interactions were observed for *Polyommatus icarus* and *Lotus corniculatus*. Just as for bees, more butterfly species and more plant–butterfly interactions were observed at three-strip-managed sites, i.e., based on averaged network-level analysis (Table 2), 87.50 interactions versus 57.75 at the regular sites (SE = 9.397, Z = −3.166, *p* = 0.001). Most other network-level parameters were found to be significantly different in the three-strip-managed margins: a higher Fisher’s alpha (SE = 2.979, Z = −2.154, *p* = 0.031), more links per species (SE = 0.097, Z = −2.577, *p* = 0.009), higher togetherness (plant level) (SE = 0.019, Z = 2.567, *p* = 0.009), a higher pollinator extinction slope (SE = 0.156, Z = −2.55, *p* = 0.01), and higher robustness for plants (SE = 0.027, Z = −2.444, *p* = 0.015) and pollinators (SE = 0.028, Z = −2.274, *p* = 0.023), as well as higher functional complementarity for plants (SE = 8.988, Z = −2.448, *p* = 0.014) and bees (SE = 7.713, Z = −2.855, *p* < 0.004). Furthermore, the parameter ‘weighted connectance’ (*p* = 0.093) was found to be borderline significant (at alpha < 0.1).

#### 3.4.3. Plant–Syrphid Visitation Networks

For syrphids, the differences in plant visitation networks for both management methods (Supplementary Figure S2) are less pronounced as compared to those for bees and butterflies. Yet the bipartite network-level analysis (Table 2) shows significantly more interactions at the three-strip-managed sites, i.e., 160.00 versus 245.50 at the regular sites (GLMM, SE = 41.42, Z = −2.064, *p* = 0.039). Moreover, fewer hoverfly species were identified for regular management, but their number was not found to be significantly different. *S. scripta* and *A. millefolium* (252 interactions) had the most interactions in the three-strip-managed margins, while in the regular ones, the highest number of interactions was found between *E. tenax* and *Daucus carota* (67 interactions). Furthermore, the parameters ‘pollinator extinction slope’ (SE = 0.3153, Z = 1.981, *p* = 0.047) and functional complementarity for pollinators (SE = 0.269, Z = −2.367, *p* = 0.018) were found to be significantly higher at the three-strip-managed sites, while other parameters were found to be borderline significant (at alpha < 0.1) or did not differ between the two management types.

## 4. Discussion

The installation of flower margins is a popular agri-environmental scheme aimed at promoting biodiversity, pollination, and pest control in farmland [60,61,62]. However, different studies have shown that outcomes after installation are highly variable [27,63,64], with management potentially being a key contributor in mitigating negative effects in an agricultural environment [65]. In this study, we broadened the assessment of three-strip management, initiated in our previous study [36], as a new management method for perennial margins in comparison with traditional (regular) phased mowing. We revealed that the new management method had a positive impact on floral diversity and the bipartite network properties of visiting pollinators, and a significant positive effect on beneficial insects, including pollinators and natural enemies.

### 4.1. Effect of Management on Abundance and Diversity Indices of Beneficial Insects

Our results show a significant positive effect on the alpha diversity of beneficial insects inhabiting perennial flower margins after three years of three-strip management. A clear effect was observed on the abundance of all pollinators (bees, syrphids, butterflies) and natural enemies, showing an increase of 44% and 50% for each group, respectively. In addition, a clear positive effect was observed on the species diversity and richness indices of pollinating bees and butterflies. Few studies compare the effects of mowing management methods on pollinator abundance and diversity indices. However, research on restoration through canopy thinning in woodlands showed a more pronounced effect on bee abundance than on diversity indices (Simpson’s diversity). Similarly, in a previous study, Parmentier (2023) [36] found that after two years, management mainly impacted bee abundance, while in our study, three years of three-strip management significantly boosted both bee richness and Shannon diversity, suggesting a cumulative positive effect on bee communities over time. Supported by differences in effect size observed between Hill numbers N1 and N2 (indices e^H’^ and D_2_, respectively) [52], our results also showed that for bees, effects were mainly observed on rarer solitary bees (categorized under the genera *Lasioglossum*, *Andrena*, *Hylaeus*, and *Megachile*) and to a lesser amount on common social bees (*Bombus* spp. and the honeybee *A. mellifera*). An explanation of this is found in the positive, cumulative effect of three-strip management, providing both food and habitat resources more effectively for less mobile species such as wild bees, with lower average flight distances [66] and rewarding floral resources located at short distances from their nests [67,68]. Common bumblebee species such as *B. pascuorum* and *B. terrestris* are still present and more numerous in farmland, and are probably less demanding regarding floral and habitat quality, and thus less responsive to different management types.

Looking at butterflies, abundance, species richness, and diversity were higher in the three-strip-managed sites. While adult butterflies also use floral nectar as a food source, non-bee pollinators such as butterflies also require suitable resources for their developing larvae, such as host plants for caterpillars [69].

For syrphids, our results are contrasting, as a significantly higher number was found in three-strip-managed sites, but not in terms of their taxonomic diversity. The contrasting effect observed between syrphids, and bees and butterflies, was also reported in a study following the yearly cumulative effects on different pollinator groups after the installation of wildflower margins in farmland [70]. The different effects observed may be explained by the opportunistic behavior of syrphids. Adult Syrphidae species feed on pollen and nectar from any available food source, and are less attached to floral diversity or habitat structures; rather, they seek the most rewarding floral patches in the landscape (see, e.g., [71]). This indicates that they are not as picky as other pollinators that fully rely on flowers for food (pollen and nectar) and habitat traits [72,73]. The larvae of syrphids do not require pollen or nectar and, in the case of Syrphidae spp., feed on aphids or plant parts, or in small water pools, making individuals in this life stage also not picky about floral quality [74].

Finally, regarding natural enemies, we mainly found a significant effect on their abundance, not on species diversity, and only a positive trend in the species richness of rarer species in the community (Hill number N1). The literature suggests that the responses of natural enemies to flower strips depend on flower strip mixtures, and flower types seem to be highly taxon-specific and vary significantly between as well as within functional groups [60]. For example, in a 3-year field study comparing the flower composition of different flower strips (annual and perennial), it was found that perennial ones showed a two- to fourfold increase in plant-dwelling spider and parasitoid wasp density, as well as higher numbers of predatory bugs and rove beetles in juvenile stages compared to annual flower strips [60]. Also, it was discussed that several factors, acting in isolation or additively, determine the attractiveness of a plant mixture to natural enemies. These factors comprise floral resource suitability (e.g., flower type), alternative prey availability, and temporal continuity [60].

### 4.2. Effect of Management on Floral Diversity and Plant–Pollinator Interactions

Based on the extended network analysis of plant–pollinator visits (Table 2), a significantly higher number of network interactions was found for all pollinator groups. In the three-strip-managed sites, floral diversity was higher, and we found more unique plant species blooming over the full season (Figure 3). We also found that after three years, some of the wild flowering species in the margins arose spontaneously, with these species also being very attractive to (mostly solitary) bees and other pollinators, e.g., *Tanacetum vulgare*, *Crepis biennis*, *R. acris*, and *R. repens*, an observation that is in agreement with other studies dealing with the improvement of wildflower mixes [19,20]. Interestingly, in relation to bees, we found that solitary bees generated more unique interactions by visiting a higher and more diverse number of plant species at three-strip-managed compared to regular-managed sites. Some plants were found to be very attractive to solitary bees, e.g., *C. capillaris*, *C. jacea* and *L. corniculatus*, which is in agreement with recent studies that investigated the attractiveness of some wildflower plants to wild bees [20,75].

For most network parameters (Table 2), a clear, significantly higher effect was found for bees and butterflies in three-strip-managed sites, while for syrphids, in most cases, only a positive trend (at alpha < 0.1) was observed. We found that the ‘Fisher’s alpha’ and the ‘number of links per species’ in the network were significantly higher for bees and butterflies (positive trend for syrphids), while ‘weighted connectance’ and ‘network H_2_’ were found to be significantly lower only for bees (positive trend for butterflies).

‘Extinction slope at pollinator level’, which is regarded as a robust network parameter [47,48], was significantly higher for butterflies and syrphids, and a positive trend was observed for bees (alpha < 0.1), suggesting that pollinators were more prone to secondary extinctions if plant taxa were eliminated from the three-strip-managed sites [53]. For plant properties in the network, interestingly, effects in different directions were observed on the ‘robustness’ and ‘functional complementarity’ (both higher in three-strip-managed sites), compared to the ‘togetherness’ of plants (lower in three-strip-managed sites). Here, functional complementarity can be seen as a mechanism by which species can divide up resources, which can facilitate co-existence and suggest that networks have greater functional integrity [76], while togetherness is a measure of the level of similarity in the distributional pattern of two species in the network [53].

The effect of such properties on the pollinators in the networks was more pronounced, with significant effects observed on their ‘robustness’ (bees, butterflies, positive trend for syrphids) and ‘functional complementarity’ (all pollinator groups). Based on the rationale behind these network parameters, this indicates that in three-strip-managed sites, total plant diversity increased, with a partitioning of the floral resources available in space and time over the full margin [47], leading to pollinator visits to different plant taxa that were more dispersed over the full margin. For pollinators, prolonging flowering activity over the season contributes to the continuous provision of food resources, and, especially in farmland, during these periods, a lower availability of floral resources is observed [61,77]. This is an important result regarding the ecosystem functioning of perennial flower margins, as a recent study also confirms that spatio-temporal complementarity of floral resources better sustains wild bee pollinators in agricultural landscapes [61]. A greater sensitivity of pollinators than plants to network robustness was also found in other studies investigating plant–pollinator network properties, and this warrants further study, as this may also have implications for predicting the relative vulnerability of plants/pollinators to extinction [47,78,79,80].

Some other network-level parameters, such as ‘overlap and decreasing fills’ (NODF), amongst others, are being reported in the literature, though we did not include them in our study (NODF was not found significant). However, contrasting conclusions regarding the consistent effects of scale are also reported for this parameter (e.g., compare Prendergast and Ollerton [47] versus Schwarzt et al. [81] in this regard). Hence, based on the significant effects found on the different network parameters selected in our study, we showed that management can have a positive impact on different properties of plant–pollinator interactions, thus influencing their feeding behavior, the feeding quality and quantity of pollinator taxa, and consequently, the reproductive state of the pollinator community, especially for rarer taxa of pollinators. In relation to the reproductive success of solitary bees, Ganser, Albrecht, and Knop [26] reported that the proximity of food resources for offspring (mainly pollen) to nesting sites seems to be more important than whether the pollen originates from plants sown in wildflower strips or from plants grown naturally along edges or in adjacent grasslands. Indeed, it has been reported that intraspecific variation in flowering phenology can modulate the precise level of spatio-temporal heterogeneity in floral resources, pollen donor density, and the pollinator interactions that a plant individual is exposed to, thereby affecting its reproduction [82]. However, as we did not investigate corbiculate pollen loads and pollen transfer between visiting pollinators and plants observed in this study, this aspect needs to be further tested.

Alongside this, significant changes in plant–pollinator interactions through adapted mowing management, as shown in our study, can have an important impact on pollination efficiency (e.g., through enhanced pollen transfer) not only for margin plants but also towards adjacent insect-pollinated crops and fruits (positive pollination spillover effect) [83].

### 4.3. Management as a Contributor to Enhanced Habitat Quality

While we did not, in this study, investigate all the relationships between elements of insect habitat (i.e., floral diversity and abundance, shelter and nesting places, mating places, and microclimates, amongst others) which are all contributing to the habitat quality of the margin, we can assume, through the application of the meandering three-strip management method (Figure 2), that different key habitat elements are more readily available over the whole season and are spatially spread over the full margin, thus generating a better habitat quality, supporting a higher and more diverse number of species and groups, respectively. One of the important habitat elements reported in the literature seems to be the presence of suitable overwintering structures, stimulating the creation of better shelter and nesting places [32,33,84]. In the case of three-strip management, old overwintering structures are always present in the margin throughout the season, which is likely to better promote the presence of pollinators [36] as well as natural enemies. In comparison, a recent study by Roudine and co-authors reported on the positive effects of uncut overwintering flower strips on the presence of natural enemies, when installed adjacent to cereal crops, as this favored the activity of ground arthropod predators. As a consequence, a reduction in aphid pest abundance and even their associated diseases was found [85]. Also, for other crop types such as fruit orchards, better-managed perennial flower margins are suitable to better supporting not only pollinators but also natural enemies, as has been shown in apple orchards, where a higher abundance of natural enemies was found to better control the rosy apple aphid *Dysaphis plantaginea*, leading to reduced fruit damage caused by this pest [86].

### 4.4. Three-Strip Management as a Nature-Based Solution to Tackle the “Flower Strip Dilemma”

Recently, Schmied and co-authors (2023) reported on the progressive flower loss observed after the installation of perennial flower margins in high-yield agricultural landscapes (but without adequate management being tested), calling it the “flower strip dilemma”, referring to the difficult choice of maintaining or reseeding the margin only a few years post installation [87]. As a solution, they suggested combined margins of remaining and frequently reseeded parts (about a one-third strip within the margin), buffering possible negative effects on either young or old flower strips regarding their specific attractiveness to different insect species and guilds, respectively. Based on our results, conducted in a similar landscape context, we still found margins to be flower-rich and diverse, attractive to different insect taxonomic groups at least up to three years post installation. This suggests that meandering three-strip management can be a valuable alternative, nature-based solution to such a dilemma. Multi-year studies over longer periods could further confirm the effects observed in this study.

In relation to the concept of combining old and new strips in the margin, it is interesting to consider their proportion. In the three-strip management method, the one third–two third mowing–no mowing aspect is not chosen randomly, hence its name, and allows for an elegant combination of sufficient new and old zones in the margin within one season and over years. In addition, the removal of clippings should control the negative effects of succession [36]. Through the continuous application of curved mowing lines, stacked as different mowing layers, a more elegant intertwining of regrown and uncut old zones is achieved. Here, still, it is very important to stress that a key element is the removal of clippings after every mowing cycle; if they are not removed, it can be expected that grasses will quickly outcompete perennial flowers and erode the margin’s biodiverse habitat quality [8,14,36].

In general, different examples, as mentioned above, stress the value of well-managed perennial flower margins not only for biodiversity but also for farmers, as high-quality margins contribute to lower damages in crops and a higher fruit yield through enhanced pollination service and pest control, and contribute to the natural regulation of the entire herbivore complex in crop fields and orchards [85,86]. This should encourage more farmers to adopt quality, nature-based schemes such as three-strip management, along with the installation of flower margins on their fields.

However, it should not be concealed that the creation of such well-established high-quality margins requires a greater effort from the farmer or contractor. They should be remunerated, for example, within the framework for payments for AEM or nature conservation funding for farmers, due to the higher effectiveness. The implementation of combinations of different ages and quality of development of flower margins in national funding programs, or at the EU policy level, should mitigate hurdles related to the implementation of improved management schemes for flower margins. In general, this can be an important basis for a more goal-oriented nature conservation plan in the agricultural landscape [87].

## 5. Conclusions

The new, nature-based three-strip management method for perennial margins, which generates variable curved mown zones staggered over time in the margins, has shown significant potential in enhancing biodiversity and supporting beneficial insects, such as pollinators and natural enemies, in agricultural landscapes.

In this study, we found a significant increase in the abundance and diversity of pollinators and natural enemies after three years of curved three-strip management, with notable improvements in species richness and species diversity indices, especially for rarer bees and pollinators in general. The new management scheme also impacted multiple properties of plant–pollinator visitation networks, increasing their strength, robustness, and complementarity, and promoting diverse and unique floral compositions that support a broader range of pollinator species. These findings suggest that three-strip management is more effective in promoting habitat quality than traditional phased (or late) mowing schemes that use uniform mown zones. It achieves this by providing a more robust and diverse supply of floral food resources and a high-quality habitat throughout the season.

Our study also indicates that three-strip management can serve as an alternative solution to tackle the “flower strip dilemma”, allowing for the maintenance of flower-diverse margins over multiple years without reseeding. Yet continued monitoring extended over years is essential to validate these findings further.

The observed benefits in this study highlight the importance of encouraging farmers to adopt new management practices along with the installation of perennial flower margins, with support through agri-environmental schemes to compensate for the additional labor and costs involved. Ultimately, this approach can enhance habitat quality in farmland, along with ecosystem services like pollination and pest control, supporting both biodiversity conservation and agricultural productivity in a future-proof agricultural landscape.

## Figures and Tables

**Table 1:** Species-level effects of management type (regular versus three-strip) on pollinators and natural enemies acting as beneficial insects. The results of the best-fitted GLMMs are shown for the assessment of the numbers and alpha diversity indices (N = abundance; S (^0^D) = species richness (Hill number N0); e^H^ (^1^D) = exponential Shannon diversity (Hill number N1); and D_2_ (^2^D) = inverse Simpson diversity (Hill number N2)) of flower-visiting pollinators (bees, hoverflies, butterflies) and natural enemies, shown as separate subgroups and pooled together. For each model, the estimate, standard error, z-value (exp(estimate)), *p*-value, and the chosen distribution are given. *p*-values in **bold** are significant at alpha = 0.05, and the asterisk indicates the significance level: * *p* < 0.05; ** *p* < 0.01; *** *p* < 0.001; Boundary effects at at alpha = 0.1 are indicated in *italic*.

	Index	Test Statistics—GLMM				Model
		Estimate	SE	Exp (Estimate)	*p*	
Bees	N	−0.320	0.129	−2.481	0.013 *	Nbinom1 (log)
	S (^0^D)	−0.360	0.113	−3.182	0.002 **	Poisson (log)
	e^H’^ (^1^D)	−0.909	0.319	−2.843	0.004 **	Gaussian
	D_2_ (^2^D)	−0.535	0.242	−2.210	0.027 *	Gaussian
Syrphids	N	−0.324	0.160	−2.021	0.043 *	Nbinom1 (log)
	S (^0^D)	−0.327	0.650	−0.504	0.727	Poisson (log)
	e^H’^ (^1^D)	−0.183	0.470	−0.401	0.689	Gaussian
	D_2_ (^2^D)	−0.226	0.379	−0.596	0.551	Gaussian
Butterflies	N	−0.875	0.289	−3.028	0.002 **	Gaussian
	S (^0^D)	−1.438	0.331	−4.338	<0.001 ***	Gaussian
	e^H’^ (^1^D)	−1.165	0.377	−3.090	0.002 **	Gaussian
	D_2_ (^2^D)	−0.955	0.318	−3.007	0.003 **	Gaussian
All pollinators	N	−0.327	0.112	−2.92	0.003 **	Nbinom1 (log)
	S (^0^D)	−2.722	0.573	−4.749	<0.001 ***	Gaussian
	e^H’^ (^1^D)	−1.311	0.436	−3.007	0.001 **	Gaussian
	D_2_ (^2^D)	−0.785	0.334	−2.351	0.019 *	Gaussian
Natural enemies	N	−0.423	0.129	−3.266	0.002 **	Nbinom2 (log)
	S (^0^D)	−0.625	0.512	−1.222	0.494	Poisson
	e^H’^ (^1^D)	−4.840	3.365	1.438	*0.105*	Gaussian
	D_2_ (^2^D)	−0.087	0.101	−0.861	0.389	Gaussian
All beneficial insects	N	−0.340	0.104	−3.28	0.001 **	Nbinom1 (log)
	S (^0^D)	−3.188	0.559	−5.703	<0.001 ***	Gaussian
	e^H’^ (^1^D)	−1.657	0.429	−3.863	<0.001 ***	Gaussian
	D_2_ (^2^D)	−1.023	0.369	−2.792	0.005 **	Gaussian

**Table 2:** Bipartite *network-level* properties per pollinator group (bee, syrphid, butterfly) and management method (regular, three-strip) after the third year of application (2023). Network properties were analyzed by constructing networks for each survey (site-scale), pooling per management type (=site) across periods and locations (n = 16); average values are reported.

	Bee		Syrphid		Butterfly	
Network Property	Regular	Three-Strip	Regular	Three-Strip	Regular	Three-Strip
N interactions	301.50 ± 112.75 **	423.25 ± 118.65 **	160.00 ± 123.99 *	245.50 ± 190.93 *	57.75 ± 18.28 **	87.50 ± 13.92 **
Fisher’s α	12.209 ± 1.171 ***	18.330 ± 3.607 ***	*10.275 ± 1.509*	*12.645 ± 4.422*	9.101 ± 6.388 *	15.520 ± 2.556 *
Links per species	1.512 ± 0.112 **	1.736 ± 0.051 **	1.502 ± 0.415	1463 ± 0.215	1.193 ± 0.211 **	1.444 ± 0.080 **
Weighted connectance	0.137 ± 0.019 **	0.111 ± 0.019 **	0.189 ± 0.041	0.165 ± 0.046	*0.168 ± 0.011*	*0.153 ± 0.017*
H_2_’	0.393 ± 0.036 **	0.301 ± 0.087 **	0.223 ± 0.115	0.253 ± 0.172	0.418 ± 0.041	0.451 ± 0.066
Extinction slope, pollinators	*2.384 ± 0.078*	*2.615 ± 0.300*	2.413 ± 0.416 *	3.037 ± 0.819 *	1.956 ± 0.492 *	2.354 ± 0.138 *
Togetherness, plants	*0.225 ± 0.053*	*0.160 ± 0.065*	*0.243 ± 0.036*	*0.195 ± 0.035*	0.179 ± 0.038 *	0.129 ± 0.025 *
Robustness, plants	0.611 ± 0.062	0.620 ± 0.063	0.545 ± 0.116	0.556 ± 0.048	0.560 ± 0.077 *	0.628 ± 0.031 *
Robustness, pollinators	0.608 ± 0.026 **	0.654 ± 0.025 **	*0.609 ± 0.041*	*0.639 ± 0.065*	0.531 ± 0.071 *	0.596 ± 0.018 *
Functional complementarity, plants	244.21 ± 130.49 ***	365.45 ± 118.72 ***	*120.79 ± 90.751*	*228.35 ± 209.51*	49.763 ± 17.195 **	71.761 ± 11.624 **
Functional complementarity, pollinators	219.99 ± 79.67 **	364.34 ± 135.46 **	113.17 ± 76.621 *	232.91 ± 219.87 *	49.404 ± 17.092 **	71.425 ± 12.825 **

Significance levels between management methods are indicated in **bold**: * α < 0.05; ** α < 0.01; *** α < 0.005; positive trends are in *italics:* α < 0.1.

## Data Availability

Data used in this study is available on request.

## Supplementary Materials

The following supporting information can be downloaded at https://www.mdpi.com/article/10.3390/insects15120953/s1, Figure S1: Plant-butterfly interactions for regular (a) versus Three-strip (b) managed margins; Figure S2: Plant-syrphid interactions for regular (a) versus Three-strip (b) managed margins; Table S1: Plant species compositions and their proportions (%) of the perennial seed mixtures sown in the paired study sites at each location; Table S2: ollinators and natural enemies observed during the transect walks over the full monitoring season in 2023, grouped per insect category, family and subdiveded per management type. File S1: Landscape analysis of study sites in locations selected for this study. An assessment of the variation in landscape metrics in the paired study sites and in relation to the management factor is given, to ensure that the landscapes of paired study sites within a defined location are similar. Land cover data were retrieved from the Biological Valua-tion Map (BVM) of Flanders, Belgium [38,39,41,68,74,88,89].
