# Supplementary material for: Biodiverse Management of Perennial Flower Margins in Farmland: Meandering Mowing by ‘Three-Strip Management’ to Boost Pollinators and Beneficial Insects
Source: Insects. 2024 Nov 30;15(12):953. doi: 10.3390/insects15120953 (PMC11677513; doi:10.3390/insects15120953)
Supplement: Supplementary file 1 [file insects-15-00953-s001.zip › Table S1. Plant species compositions and their proportions in the perennial seed mixtures.pdf]

Suppl. Table 1.

Plant species compositions and their proportions (%) of the perennial seed mixtures sown in the paired study sites at each location (location 1-5 and abbreviations of study sites in the header: Her = herzele, Mal = maldegem, Meul = Meulebeke, StLAUR = Sint-Laureins, Wer = Wervik).

**Location 4: HER 42 & 40**	**Proportion (%)**	**Location 3, 5: HER 19 & 22; HER38A & 38B**	**Proportion (%)**	**Location 1, 2: MAL & MEUL; StLAUR & WER**	**Proportion (%)**
Centaurea cyanus	18	*Festuca rubra*	50	*Daucus carota*	15
Daucus carota	14	*Avena sativa*	10	*Leucanthemum vulgare*	15
Centaurea jacea	14	*Phleum pratense*	5	*Medicago lupulina*	14
Lotus corniculatus	14	*Poa pratensis*	5	*Lotus corniculatus*	13,5
Achillea millefolium	8	*Festuca arundinacea*	5	*Anthriscus sylvestris*	12
Tanacetum vulgare	8	*Dactylis glomerata*	5	*Trifolium pratense*	10
Matricaria chamomilla	6	*Festuca pratensis*	5	*Prunella vulgaris*	9
Trifolium pratense	6	*Centaurea jacea*	3	*Ranunculus acris*	7
Trifolium repens	6	*Plantago lanceolata*	3	*Achillea millefolium*	3
Crepis capillaris	6	*Daucus carota*	3	*Hypericum perforatum*	0,5
		Centaurea cyanus	2	*Veronica Chamaedris*	0,5
		Medicago sativa	2	*Berteroa incana*	0,5
		Trifolium pratense	2		
		Leucanthemum vulgare	1		
		Malva sylvestris	1		
