# Supplementary material for: Biodiverse Management of Perennial Flower Margins in Farmland: Meandering Mowing by ‘Three-Strip Management’ to Boost Pollinators and Beneficial Insects
Source: Insects. 2024 Nov 30;15(12):953. doi: 10.3390/insects15120953 (PMC11677513; doi:10.3390/insects15120953)
Supplement: Supplementary file 1 [file insects-15-00953-s001.zip › Table S2. Observations of pollinators and natural enemies during the study.pdf]

Suppl. Table 2.

Pollinators and natural enemies observed during the transect walks over the full monitoring season in 2023, grouped per insect category, family and subdiveded per management type.

A. **pollinators**

	Three-strip management	Regular management
**Pollinators**	**3168**	**2199**
**Andrenidae**	**15**	**10**
Andrena flavipes	4	2
Andrena haemorrhoa	1	
Andrena humilis	3	4
Panurgus calcaratus	7	4
**Apidae**	**1440**	**1086**
Apis mellifera	235	295
Bombus campestris		3
Bombus hortorum	4	7
Bombus hypnorum	7	10
Bombus jonellus	3	
Bombus lapidarius	328	220
Bombus pascuorum	725	474
Bombus pratorum		1
Bombus sylvestris	1	
Bombus terrestris complex	137	76
**Colletidae**	**48**	**58**
Colletes daviesanus	40	58
Colletes similis	1	
Hylaeus cornutus	7	
**Halictidae**	**141**	**47**
Halictus scabiosae	31	11
Halictus tumulorum	46	14
Lasioglossum brevicorne	5	
Lasioglossum calceatum	6	
Lasioglossum fulvicorne	2	
Lasioglossum leucozonium	5	
Lasioglossum malachurum	15	14
Lasioglossum morio	5	
Lasioglossum pauxillum	3	3
Lasioglossum sabulosum	2	

Lasioglossum sextrigatum	1	
Lasioglossum villosulum	15	5
Sphecodes gibbus	4	
Sphecodes spinulosus	1	
**Hesperiidae**	**41**	**11**
Ochlodes sylvanus	29	11
Thymelicus lineola	11	
Thymelicus lineola/sylvestris	1	
**Lycaenidae**	**123**	**93**
Aricia agestis	16	15
Polyommatus icarus	91	76
Lycaena phlaeas	16	2
**Megachilidae**	**51**	**15**
Anthidium manicatum	1	
Chelostoma campanularum	1	
Chelostoma florisomne		1
Chelostoma rapunchuli	2	
Heriades truncorum	6	
Megachile centuncularis	13	5
Megachile ligniseca	2	1
Megachile rotundata		2
Megachile versicolor		4
Megachile willughbiella	24	1
Stelis breviuscula	1	1
Trachusa byssina	1	
**Melittidae**	**19**	**3**
Dasypoda hirtipes	19	
Melitta leporina		3
**Nymphalidae**	**143**	**84**
Aglais io	15	9
Araschnia levana	2	4
Coenonympha pamphilus	5	2
Maniola jurtina	96	57
Pararge aegeria	1	
Polygonia c-album		1
Pyronia tithonus	6	5
Vanessa atalanta	17	3

Vanessa cardui	1	3
**Pieridae**	**142**	**116**
Aporia crataegi	1	
Pieris brassicae	3	3
Pieris napi	20	5
Pieris rapae	118	108
**Syrphidae**	**1005**	**676**
Cheilosia bergenstammi		1
Cheilosia pagana		1
Episyrphus balteatus	37	40
Eristalinus sepulchralis		1
Eristalis arbustorum	134	79
Eristalis horticola	1	
Eristalis nemorum	51	60
Eristalis tenax	232	177
Eumerus strigatus	1	
Eupeodes corollae	13	14
Eupeodes latifasciatus	1	
Eupeodes luniger		1
Helophilus pendulus	1	3
Helophilus trivittatus	2	10
Melanostoma mellinum	17	9
Melanostoma scalare	1	
Myathropa florea	11	9
Platycheirus albimanus		3
Platycheirus scutatus	4	
Pyrophaena rosarum		6
Rhingia campestris	2	
Sphaerophoria batava	1	28
Sphaerophoria philanthus	11	10
Sphaerophoria rueppelli	5	3
Sphaerophoria scripta	463	194
Syritta pipiens	11	20
Syrphus ribesii	3	6
Syrphus vitripennis	1	
Volucella zonaria		1
Xanthogramma pedissequum	2	

B. **Natural ennemies***

*** Selection of the species was based on positive predation traits on crop pests as reported in the sources mentioned in brackets**

	Driebandbeheer	Regulier beheer
**Natural enemies**	**699**	**432**
**Asilidae(1)**	**1**	
Dioctria cothurnata	1	
**Cantharidae(2)**	**25**	**27**
Cantharis flavilabris		7
Cantharis nigra		2
Rhagonycha fulva	25	18
**Chrysopidae**	**2**	**1**
Chrysoperla carnea(3)	2	1
**Coccinellidae(4)**	**48**	**39**
Coccinella magnifica	1	
Coccinella septempunctata	34	27
Harmonia axyridis	7	12
Propylea quatuordecimpunctata	3	
Psyllobora vigintiduopunctata	3	
**Crabronidae(5)**	**22**	**4**
Cerceris arenaria	1	
Cerceris quadricincta	8	
Lindenius albilabris	11	4
Oxybelus uniglumis	2	
**Ichneumonidae(6)**	**28**	**21**
Ichneumonidae spp.	28	21
**Miridae**	**7**	**2**
Deraeocoris ruber(7)	7	2
**Syrphidae**	**559**	**308**
Episyrphus balteatus(3)	37	40
Eupeodes corollae(8)	13	14
Eupeodes latifasciatus(8)	1	
Eupeodes luniger(8)		1
Melanostoma mellinum(3)	17	9
Melanostoma scalare(9)	1	

Platycheirus albimanus[(10)]		3
Platycheirus scutatus[(11)]	4	
Sphaerophoria batava[(11)]	1	28
Sphaerophoria philanthus[(11)]	11	10
Sphaerophoria rueppelli[(8)]	5	3
Sphaerophoria scripta[(3)]	463	194
Syrphus ribesii[(3)]	3	6
Syrphus vitripennis[(3)]	1	
Xanthogramma pedissequum[(12)]	2	
**Vespidae**	**7**	**30**
Ancistrocerus parietum[(13)]	1	13
Polistes dominula[(14)]	2	6
Vespula germanica[(15)]	4	11

Sources: (1) Wegensteiner et al., 2015; (2) Pelletier & Hébert, 2014; (3) Vandereycken et al., 2015; (4) Krinsky, 2002; (5) Marchiori, 2023; (6) Dreistadt, 2014; (7) Dicker, 1952; (8) Pekas et al., 2020; (9) Wilkinson & Rotheray, 2017; (10) Jansen & Warnier, 2004; (11) Almohamad et al., 2009; (12) Sivell & Sivell, 2022; (13) Archer, 2011; (14) McGruddy et al., 2021; (15) Pusceddu et al., 2018.
