# Supplementary material for: Biodiverse Management of Perennial Flower Margins in Farmland: Meandering Mowing by ‘Three-Strip Management’ to Boost Pollinators and Beneficial Insects
Source: Insects. 2024 Nov 30;15(12):953. doi: 10.3390/insects15120953 (PMC11677513; doi:10.3390/insects15120953)
Supplement: Supplementary file 1 [file insects-15-00953-s001.zip › File S1. Land cover analysis of study sites.pdf]

Supplementary material

Land cover analysis of study sites

Biodiverse management of perennial flower margins in farmland: meandering mowing by 'three-strip management' to boost pollinators and beneficial insects

Laurian Parmentier [1,2,*], Hannah Vanderstappen [1,2] and Geert Haesaert [2]

[1] Ghent University, Faculty of Bioscience Engineering, Department of Plants and Crops, Agrozoology Lab, Coupure Links 653, 9000 Ghent, Belgium
[2] Ghent University, Faculty of Bioscience Engineering, Department Plants and Crops, Valentin Vaerwyckweg 1, 9000 Gent

* Correspondence: laurian.parmentier@ugent.be

To evaluate the effect of the two different management methods, i.e. the novel three-strip management (3S) versus sinus management (S) on pollinators and natural enemies, we used a coupled study site design in selected locations situated in anthropogenic landscapes with different metrics (including agricultural, semi-natural and rural elements) situated in Flanders, Belgium. In each location the goal was to select two identical study sites for installing flower margins situated in an equal landscape. To ensure landscapes equally supported beneficial insect populations (i.e., in this study pollinators and natural enemies) we performed an analysis of landscape metrics of each location and study site. As most landscape metrics are evolving slowly in the given study period of 3 years, we performed the landscape analysis preliminary to the start of the study in 2020. Only study sites with equal landscape metrics within a given location were selected in our paired study design; on the contrary, differences in landscape metrics between locations (but not between study sites) means landscape variation and could be included as random variation in the study.

A landscape analysis was performed within a radius of 750 m from the center of the study sites, based on average flying distance of recorded insect groups [1-3]. Land cover data was retrieved from the Biological Valuation Map (BVM) of Flanders [4] and analyzed in QGis [5]. **Figure S1.1** gives an overview of all paired study sites per location. BVM categories were grouped in seven land cover categories, based on the resources they provide for pollinators and beneficial insects. The following land cover categories were identified: (1) "Seminatural positive", encompassing all semi-natural habitats (permanent grasslands, woodlands, positive linear elements) and small landscape elements (SLE) which can provide suitable food or nesting places, (2) "Semi-natural neutral", all semi-natural elements that do not provide food or nesting (e.g. ponds, temporary species-poor quality grasslands and negative linear elements…), (3) "Urban", defined as the percentage of build-up, industrial areas and roads, (4) "Waterbodies", grouping all water surfaces (water basins, rivers creeks,..) (5) "Acres", encompassing all intensively used agricultural areas, excluding insect-pollinated crops (grain, potatoes, maize, beets,…), (6) Orchards, describing all plantations of low or high stem fruit trees (e.g. apples, pear, sweet cherry,…) and (7) others, encompassing undefined elements in the land cover maps (minority of the full land cover). **Table A1.1** is giving the percentage of land cover of different categories per study site.

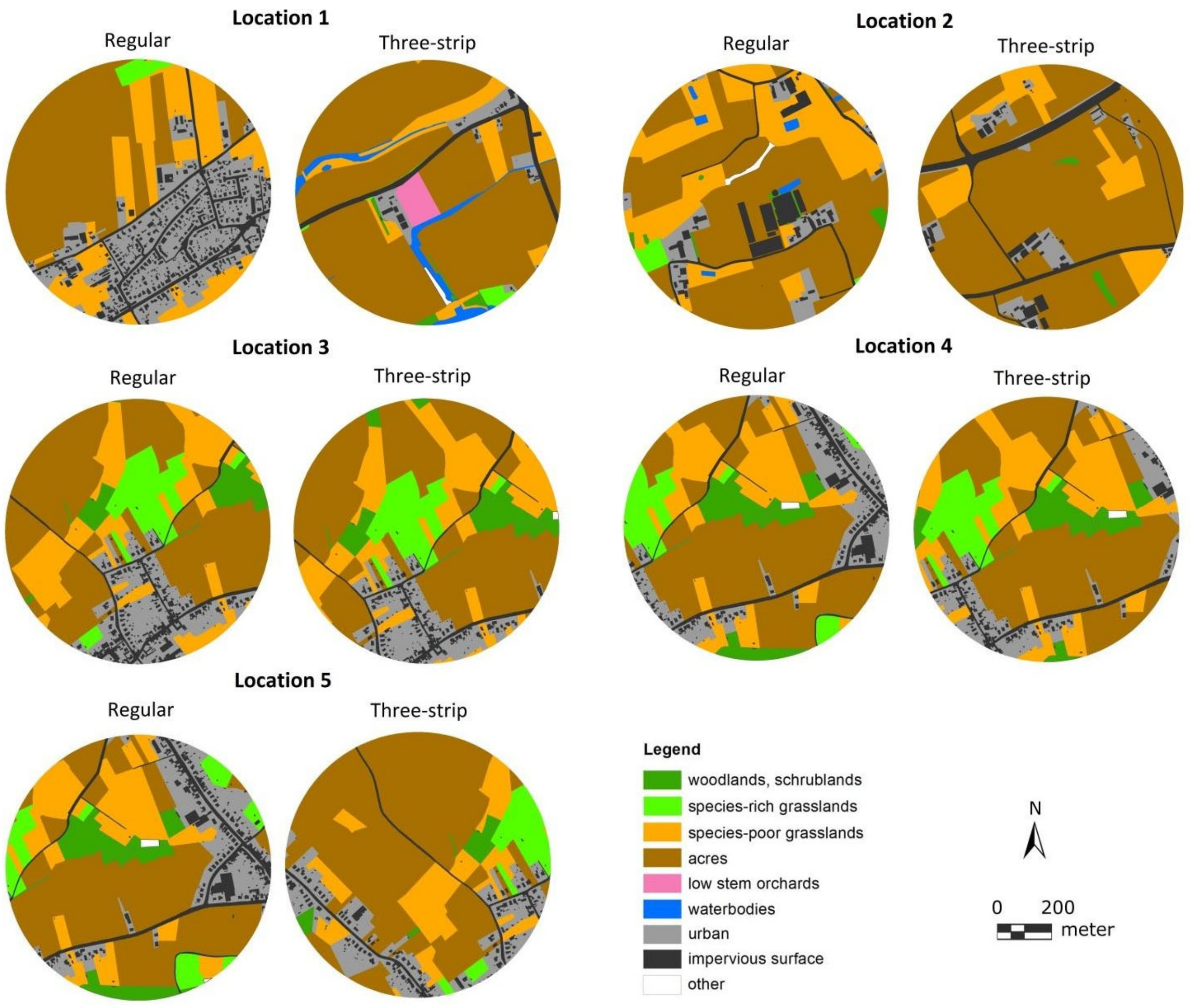

Figure A1.1 Landscape cover by categories of selected paired study sites (Regular – Three-strip) in all five locations

Table A1.1: Percentage of land cover per study site and location

Location	Site	Management	Grass_rich	Grass_poor	Urban	Acres	Orchard	SLE	Waterbodies	Other
HER40_42	HER40	3S	5.91	19.43	14.27	42.95	0.00	8.43	0.00	9.00
HER40_42	HER42	R	6.14	14.69	14.58	56.82	0.00	2.34	0.00	5.42
HER38A_38B	HER38R	R	6.73	19.57	13.48	44.06	0.00	7.97	0.00	8.19
HER38A_38B	HER38B	3S	8.38	23.18	10.58	45.17	0.00	7.37	0.00	5.32
HER19_22	HER19	R	8.93	20.47	12.57	46.19	0.00	5.16	0.00	6.68
HER19_22	HER22	3S	8.38	21.79	10.23	47.60	0.00	6.84	0.00	5.16
Meul_Werv	Meul	R	1.30	26.76	6.76	54.44	0.00	1.05	1.05	8.64
Meul_Werv	Werv	3S	0.00	11.96	5.41	72.80	0.00	0.70	0.00	9.13
Dam_StLaur	Dam	3S	1.45	21.14	26.97	36.60	0.00	0.00	0.00	13.85
Dam_StLaur	StLaur	R	0.90	9.13	4.48	73.94	2.46	1.00	4.01	4.08

To verify landscape metrics of the study site design, the land cover data of the selected locations and study sites situated in East- and West Flanders were transformed into a similarity matrix based upon Euclidean-distance and a Permutational multivariate analysis of variance (PERMANOVA) was performed with the Adonis function in the R package Vegan [6]. We tested if the land cover matrix was different in relation to the factor management (two levels, Three-strip, Regular) and the factor location (five levels).

The results show that study sites (factor management) within selected locations are not different ($F_{5,10}$ = 0.963, p = 0.489), and locations are situated in landscapes with equal metrics ($F_{4,5}$ = 0.981, p = 0.565). Thus, the selected coupled study site design is in agreement with our premise. **Figure A1.2** represents the landscape analysis through an NMDS plot using the ggplot2 package in the R [7]. Circles in blue and red colour represent ordihulls grouping the study sites acciring to assignment of management treatments, i.e., three-strip management (3B) and regular manegment (R), respectively, together. As visually seen, the ordihulls are strongly overlapping showing that a minimal difference of landscape metrics exists between the paired study sites in this study.

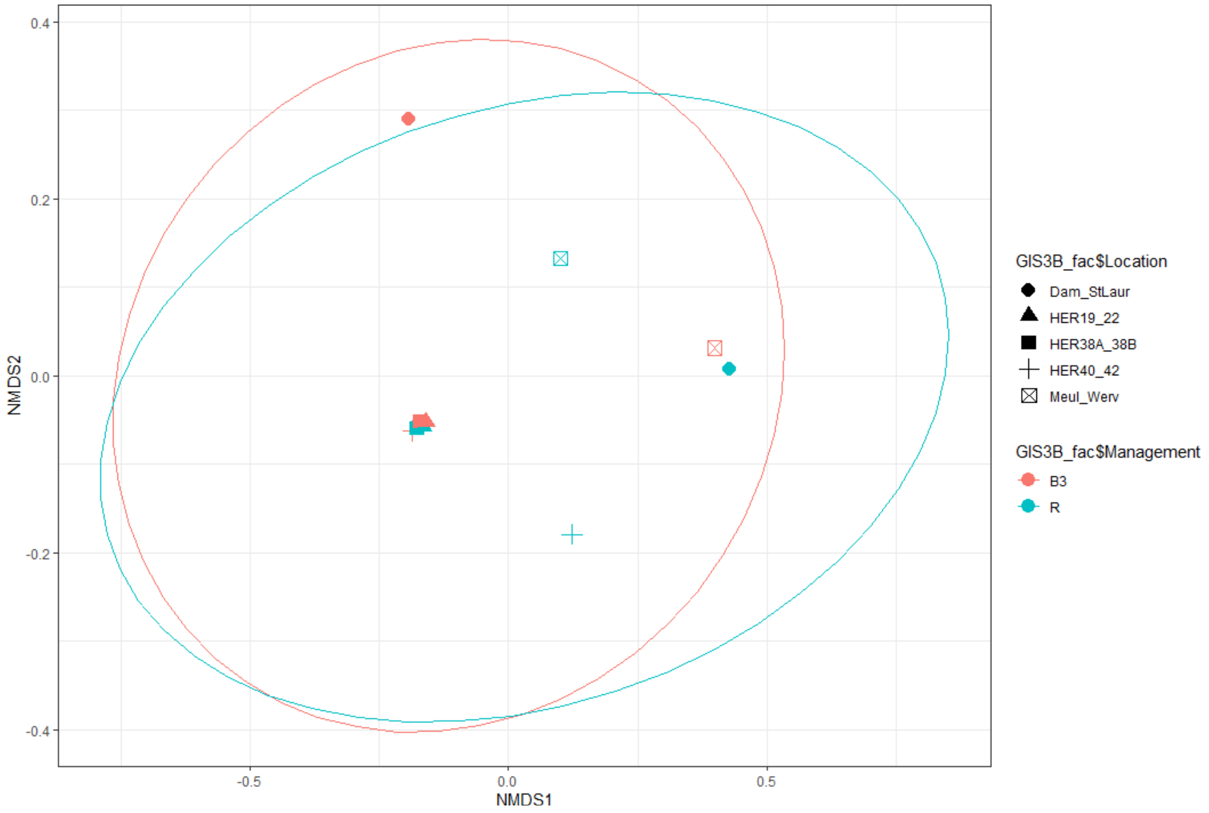

Figure A1.2. NMDS plot representing PERMANOVA analysis of land cover of all five locations with paired study sites attributed to either three-strip management (B3) or Regular management (R).

References

1. Walther-Hellwig, K.; Frankl, R. Foraging distances of *Bombus muscorum*, *Bombus lapidarius*, and *Bombus terrestris* (Hymenoptera, Apidae). *Journal of Insect Behavior* **2000**, *13*, 239-246, doi:Doi 10.1023/A:1007740315207.
2. Peeters, T.M.J.; Nieuwenhuijsen, H.; Smit, J.; van der Meer, F.; Raemakers, I.P.; Heitmans, W.R.B.; van Achterberg, C.; Kwak, M.; Loonstra, A.J.; de Rond, J.; et al. *De Nederlandse bijen (Hymenoptera: Apidae s.l.). - Natuur van Nederland*; Leiden, The Netherlands, 2012.
3. Reemer, M. Britain's Hoverflies: An Introduction to the Hoverflies of Britain. *Syst. Entomol.* **2014**, *39*, 196-196, doi:10.1111/syen.12041.
4. De Saeger, S.; Guelinckx, R.; Oosterlynck, P.; Erens, R.; Hennebel, D.; Jacobs, I.; Van Oost, F.; Van Dam, G.; Van Hove, M.; Wils, C.; et al. Biologische Waarderingskaart en Natura 2000 Habitatkaart, uitgave 2016. **2016**.
5. QGIS_Development_Team. QGIS Geographic Information System. Available online: (accessed on
6. Oksanen, J.; Blanchet, F.G.; Kindt, R.; Legendre, P.; Minchin, P.R.; O'Hara, R.B.; Simpson, G.L.; Solymos, P.; Henry, M.; Stevens, H.; et al. Community Ecology Package 'Vegan'. **2016**.
7. Villanueva, R.A.M.; Chen, Z.J. ggplot2: Elegant Graphics for Data Analysis, 2nd edition. *Measurement-Interdisciplinary Research and Perspectives* **2019**, *17*, 160-167, doi:10.1080/15366367.2019.1565254.
