# Supplementary figures and images for: Biodiverse Management of Perennial Flower Margins in Farmland: Meandering Mowing by ‘Three-Strip Management’ to Boost Pollinators and Beneficial Insects
Source: Insects. 2024 Nov 30;15(12):953. doi: 10.3390/insects15120953 (PMC11677513; doi:10.3390/insects15120953)

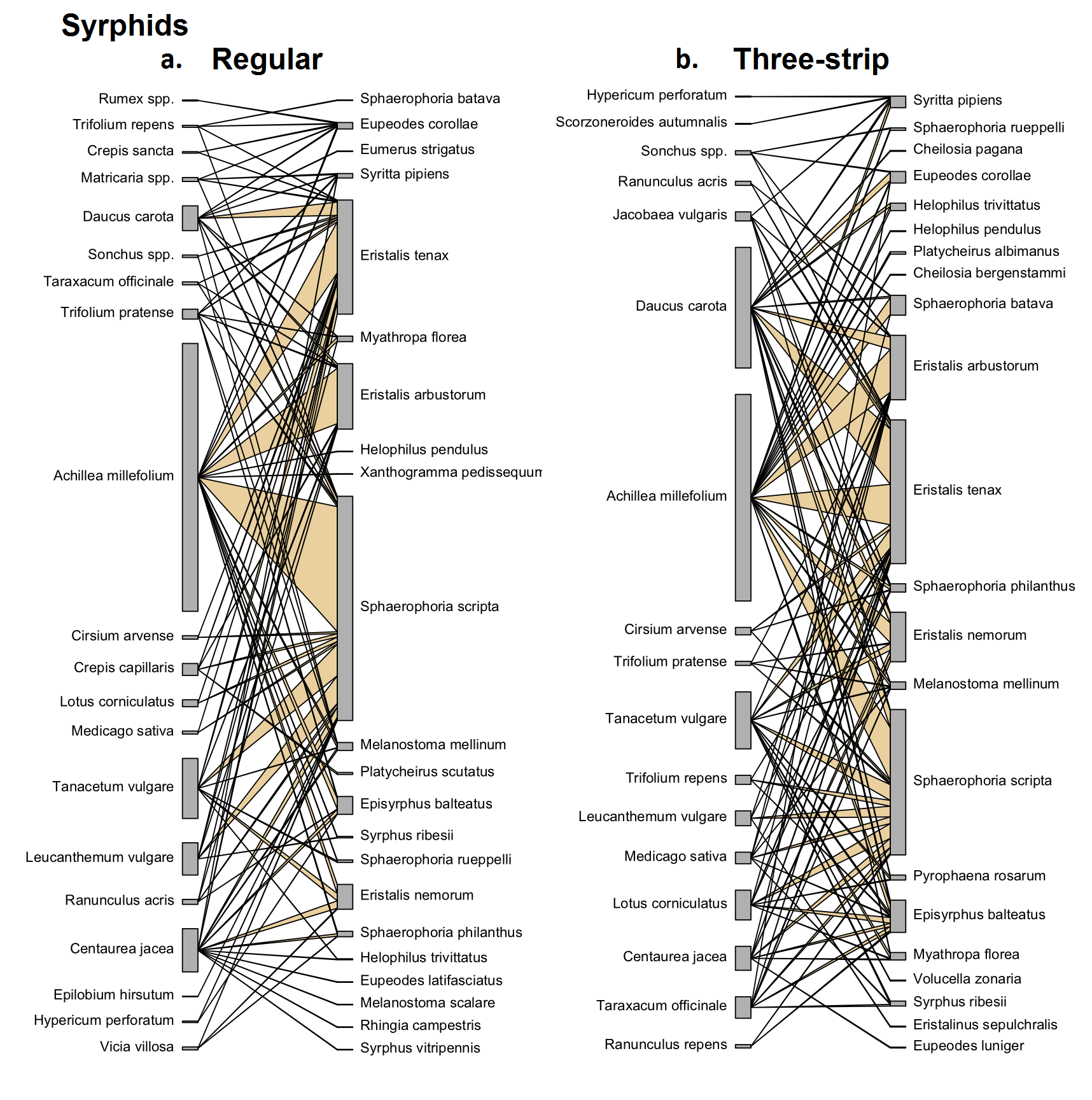

Supplement: Supplementary file 1 [file insects-15-00953-s001.zip › Figure S2. Plant-syrphid interactions.tif]

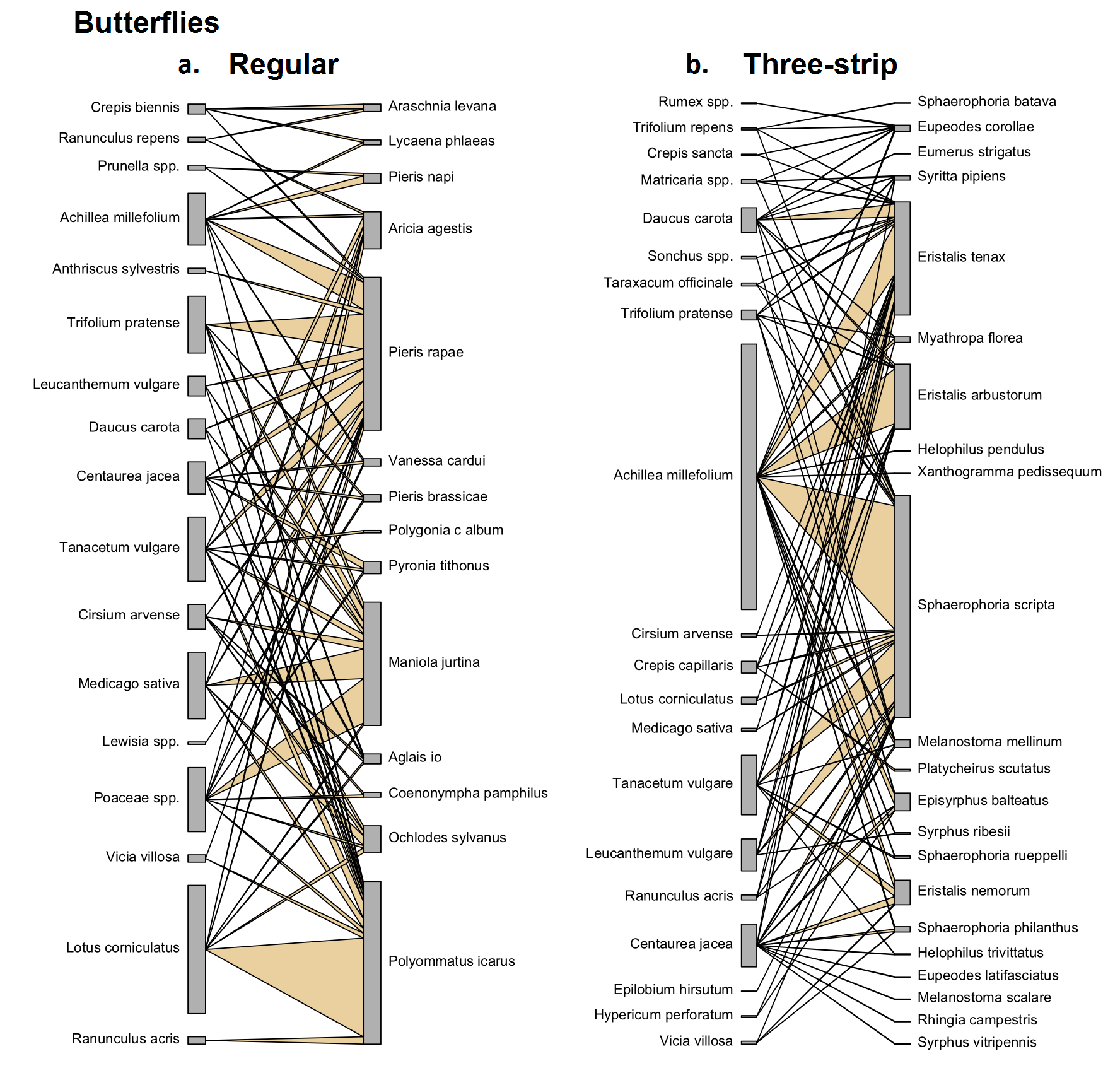

Supplement: Supplementary file 1 [file insects-15-00953-s001.zip › Figure S1. Plant-butterfly interactions.tif]
